# Replicative Endothelial Cell Senescence May Lead to Endothelial Dysfunction by Increasing the BH2/BH4 Ratio Induced by Oxidative Stress, Reducing BH4 Availability, and Decreasing the Expression of eNOS

**DOI:** 10.3390/ijms25189890

**Published:** 2024-09-13

**Authors:** Ignacio Hernandez-Navarro, Laura Botana, Javier Diez-Mata, Laura Tesoro, Beatriz Jimenez-Guirado, Claudia Gonzalez-Cucharero, Nunzio Alcharani, Jose Luis Zamorano, Marta Saura, Carlos Zaragoza

**Affiliations:** 1 Unidad Mixta de Investigación Cardiovascular Universidad Francisco de Vitoria, Hospital Universitario Ramón y Cajal (IRYCIS), 28034 Madrid, Spain; naxete1992@gmail.com (I.H.-N.); laura.botana@ufv.es (L.B.); jdiezmata@gmail.com (J.D.-M.); laura.tesoro@ufv.es (L.T.); b.jimenezguirado@gmail.com (B.J.-G.); claudia311099@gmail.com (C.G.-C.); nunzio.alcharani@ufv.es (N.A.); 2 Centro de Investigación Biomédica en Red de Enfermedades Cardiovasculares (CIBERCV), Instituto de Salud Carlos III (ISCIII), 28029 Madrid, Spain; zamorano@secardiologia.es (J.L.Z.); marta.saura@uah.es (M.S.); 3 Facultad de Ciencias Experimentales, Universidad Francisco de Vitoria, 28223 Madrid, Spain; 4 Facultad de Medicina, Universidad Francisco de Vitoria, 28223 Madrid, Spain; 5 Departamento de Cardiología, Hospital Universitario Ramón y Cajal (IRYCIS), 28034 Madrid, Spain; 6 Unidad de Fisiología, Departamento de Biología de Sistemas, Universidad de Alcalá (IRYCIS), 28871 Alcala de Henares, Spain

**Keywords:** endothelial senescence (ES), nitric oxide (NO), endothelial nitric oxide synthase (eNOS) uncoupling, tetrahydrobiopterin (BH4), oxidative stress, inflammation, Cyclophilin A (CypA), extracellular matrix metalloprotease inducer (EMMPRIN)

## Abstract

Vascular aging is associated with the development of cardiovascular complications, in which endothelial cell senescence (ES) may play a critical role. Nitric oxide (NO) prevents human ES through inhibition of oxidative stress, and inflammatory signaling by mechanisms yet to be elucidated. Endothelial cells undergo an irreversible growth arrest and alter their functional state after a finite number of divisions, a phenomenon called replicative senescence. We assessed the contribution of NO during replicative senescence of human aortic (HAEC) and coronary (CAEC) endothelial cells, in which accumulation of the senescence marker SA-β-Gal was quantified by β-galactosidase staining on cultured cells. We found a negative correlation in passaged cell cultures from P0 to P12, between a reduction in NO production with increased ES and the formation of reactive oxygen (ROS) and nitrogen (ONOO^−^) species, indicative of oxidative and nitrosative stress. The effect of ES was evidenced by reduced expression of endothelial Nitric Oxide Synthase (eNOS), Interleukin Linked Kinase (ILK), and Heat shock protein 90 (Hsp90), alongside a significant increase in the BH2/BH4 ratio, inducing the uncoupling of eNOS, favoring the production of superoxide and peroxynitrite species, and fostering an inflammatory environment, as confirmed by the levels of Cyclophilin A (CypA) and its receptor Extracellular Matrix Metalloprotease Inducer (EMMPRIN). NO prevents ES by preventing the uncoupling of eNOS, in which oxidation of BH4, which plays a key role in eNOS producing NO, may play a critical role in launching the release of free radical species, triggering an aging-related inflammatory response.

## 1. Introduction

Endothelial dysfunction (ED) is an early indicator of vascular disease and can precede the development of several cardiovascular complications, including hypertension, atherosclerosis, and coronary artery disease. The impaired endothelium loses its ability to regulate vascular tone, which can lead to increased vascular resistance, a reduced blood supply to tissues, oxidative stress, and ultimately, cardiovascular events [1].

Endothelial nitric oxide (NO) maintains vascular tone and prevents endothelial dysfunction (ED) by promoting vasodilation and preventing platelet aggregation and monocyte adhesion [2]. A significant contributor to this process is Interleukin-linked kinase (ILK), a Ser/Thr kinase that keeps eNOS bound with Hsp90, which is necessary for NO production in vascular endothelial cells [3].

Cellular senescence is defined by the irreversible activation of a cell cycle arrest, induced by causes that include increased oxidative stress, and related factors [4,5,6]. In this regard, ILK plays a crucial role in preventing ES by promoting eNOS-induced production of NO. ILK achieves this effect by ensuring the coupling of eNOS with Hsp90, a necessary condition for eNOS-induced NO production in vascular endothelial cells. This process prevents superoxide ion generation, which may contribute to increased oxidative stress, a key factor related to ES [7,8].

Tetrahydrobiopterin (BH4) is a pivotal cofactor that enables eNOS to produce NO, by serving as an electron donor for heme-bound oxygen and enabling the oxidation of L-Arg to L-citrulline, which releases NO [9]. When BH4 oxidizes to BH2, eNOS starts producing superoxide anion instead, leading to increased oxidative stress. Overproduction of reactive oxygen species (ROS) is a central factor in triggering inflammatory-related complications. Notably, Cyclophilin A (CyPA) is a primary ROS-induced factor that amplifies inflammatory responses within the vascular system. Indeed, it is widely recognized that CypA serves as the endogenous ligand for Extracellular Matrix Metalloproteinase Inducer (EMMPRIN), setting off a series of inflammatory events that includes matrix metalloproteinase-induced extracellular matrix degradation, followed by smooth and endothelial cell necrosis, ultimately contributing to atheroma plaque progression.

Recent human studies have shown a strong correlation between an excess of BH2 and aging [10]. Additionally, the rate-limiting enzyme in the biosynthesis of BH4, GTP cyclohydrolase (GTPCH), plays a key role in aging-related complications. GTPCH deficiency is associated with peripheral and microvascular dysfunction [11], while its overexpression restores endothelial NO production in aging mice [12].

A reduction in eNOS-induced NO production and diminished availability of BH4 are hallmarks of both ES and ED [13]. NO is a critical player in both processes by preventing the release of reactive oxygen and nitrogen species, mitochondrial dysfunction, inhibiting the onset of inflammation, and inhibiting ED [14]. Therefore, further investigation is urgently needed to determine if nitric oxide’s (NO’s) ability to prevent endothelial dysfunction (ED) in vascular aging relies on its impact on endothelial cell senescence. These findings could significantly advance our understanding of cardiovascular aging and the development of potential preventive strategies.

To assess whether ES plays a key role in the onset of vascular endothelial dysfunction, we conducted a replicative senescence assay in primary human aortic and coronary endothelial cell cultures to evaluate the association between endothelial senescence and the mechanisms that may lead to reduced NO bioavailability. Our data could offer valuable insights into the correlation between aging and the occurrence of prevalent cardiovascular diseases, such as atherosclerosis and coronary artery disease, potentially distinguishing between vascular vs. chronological age.

## 2. Results

### 2.1. ES Induced the Expression of Inflammatory Markers in Endothelial Cells

**Figure 1:**
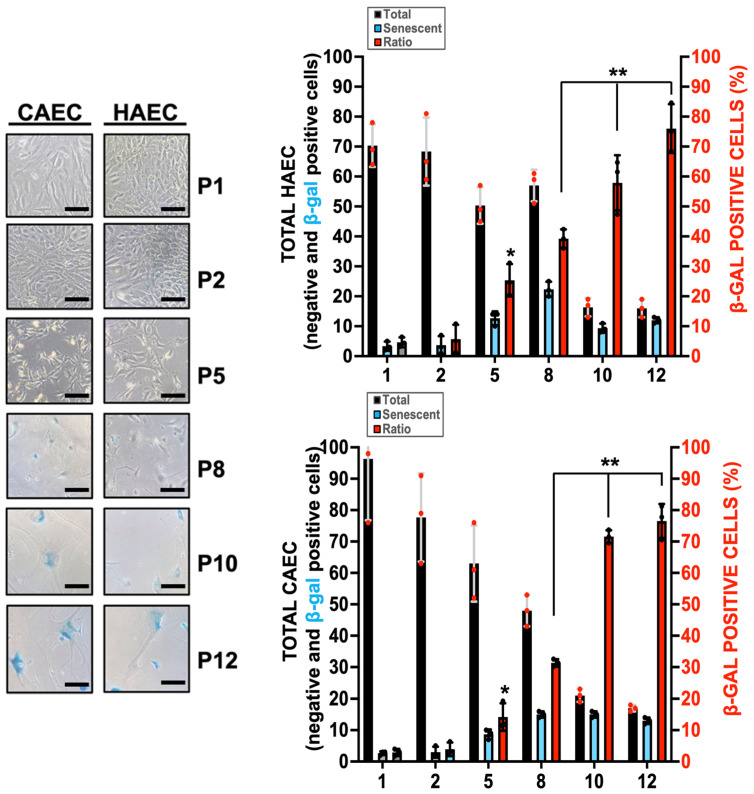
Passage-induced senescence in cultured HAEC and CAEC. Representative Beta-Galactosidase staining of cultured HAEC and CAEC from Passage P1 to P12. N = 3 by triplicate, Mean ± SD. * *p* < 0.05 P5 vs. P1. ** *p* < 0.01 P8, P10, P12 vs. P1 (HAEC&CAEC). Scale bars, 50 µm.

To more accurately reproduce the human condition in vitro, we opted for using human primary aortic (HAEC) and coronary (CAEC) endothelial cells, assessing ES in cultures spanning passages P1 through P12, in a replicative senescence assay by using Beta-Galactosidase (Beta-Gal) staining as an indicator. Our findings unveiled a distinct negative correlation between the population of living cells and the occurrence of Beta-Gal-positive endothelial cells. The rise in ES became notably pronounced beginning at P5 for both cell types (Figure 1), underscoring the escalating manifestation of endothelial aging with consecutive cell passages.

**Figure 2:**
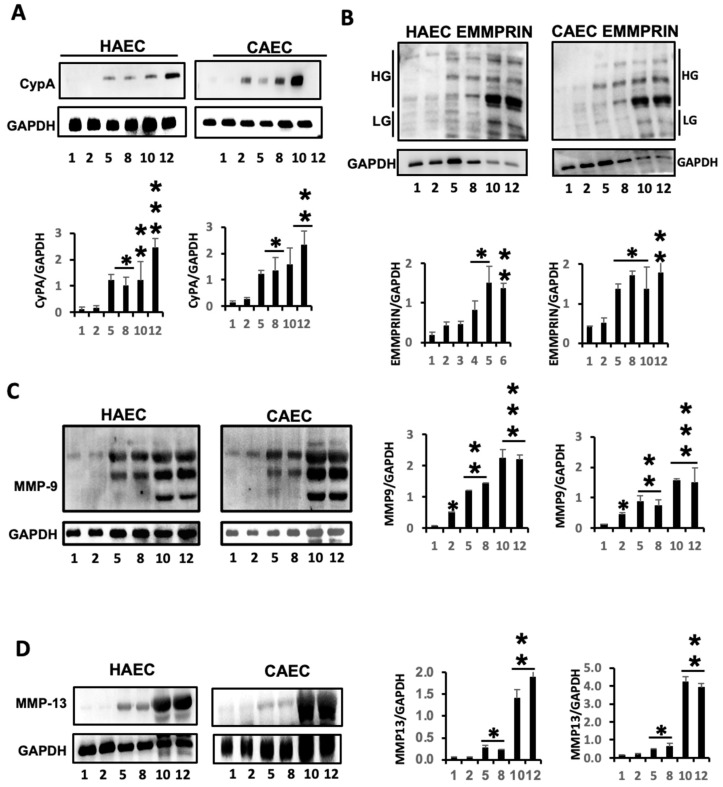
ES promotes the expression of inflammatory markers in ECs. Representative immunoblot detection of Cyclophilin A (**A**) and its receptor EMMPRIN (high glycosylated (HG) and low glycosylated (LG) forms) (**B**). N = 3 by triplicate. Mean ± SD * *p* < 0.01, ** *p* < 0.001, *** *p* < 0.0001 P1 vs. selected passages. (**C**,**D**) Representative expression of matrix metalloproteinases MM9 (**C**) and MMP13 (**D**) in the same cells. N = 3 by triplicate. Mean ± SD. * *p* < 0.05, ** *p* < 0.01, *** *p* < 0.001 P1 vs. indicated passages.

The levels of both Cyclophilin A (CypA) (Figure 2A) and EMMPRIN (Figure 2B) inflammatory markers were notably upregulated in senescent endothelial cells. Particularly noteworthy was the elevated presence of highly glycosylated forms of EMMPRIN (HG-EMMPRIN), a major factor associated with EMMPRIN-mediated MMP activation, especially evident in later cell passages. Significantly, two pivotal MMPs linked to inflammatory-related cardiovascular diseases, MMP-9 and MMP-13 [15], exhibited marked elevations in response to endothelial senescence (Figure 2C,D), suggesting that the inflammatory response associated with endothelial senescence (ES) may be initiated, at least in part, by events that trigger oxidative stress.

### 2.2. The Levels of eNOS Were Markedly Reduced in Response to Replicative ES, and Promoted eNOS Cofactor BH4 Deprivation

**Figure 3:**
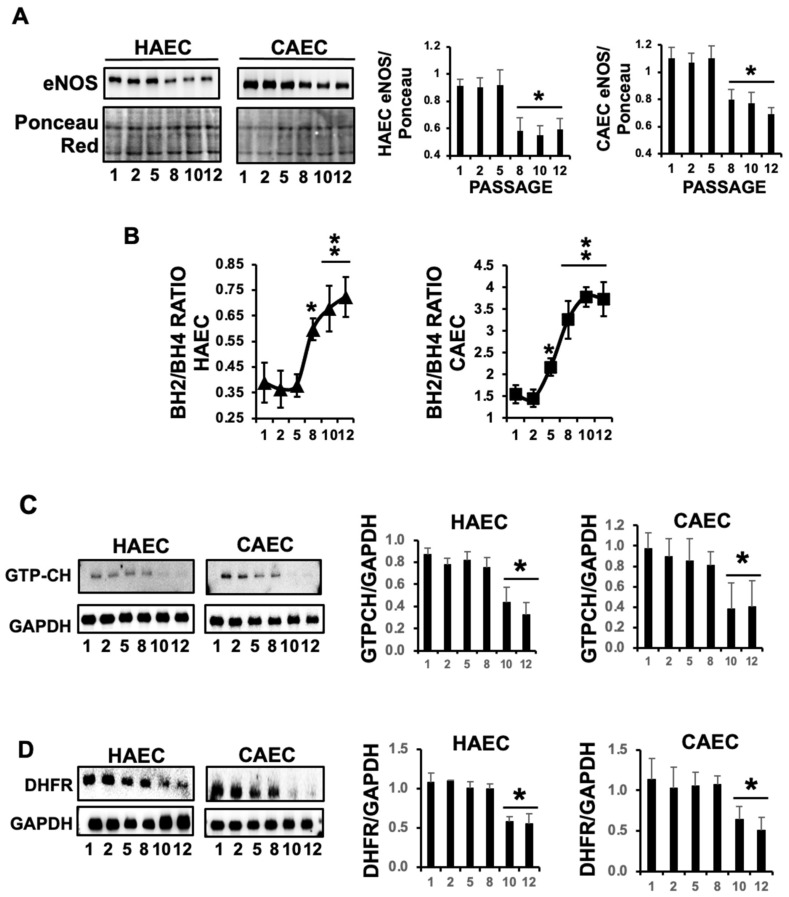
ES reduces the expression of eNOS and promotes oxidation of BH4 in HAEC and CAEC. (**A**) Representative expression of eNOS from P1 to P12 (N = 3 by triplicate. Mean ± SD. * *p* < 0.01 selected passages vs. P1). (**B**) Representative ratio BH2/BH4 in cultured HAEC and CAEC from P1 to P12 (N = 3 by triplicate. Mean ± SD. * *p* < 0.01, ** *p* < 0.001). (**C**,**D**) Representative expression of GTP cyclohydrolase and dihydrofolate reductase (DHFR), respectively, in cultured HAEC and CAEC from P1 to P12 (N = 3 by triplicate. Mean ± SD. * *p* < 0.01 vs. selected passages).

ED is defined by the loss of vessel-relaxing ability induced by the endothelial layer, in which endothelial NO plays a pivotal role. In light of this, our initial investigation focused on assessing the regulation of NO signaling in response to ES within HAEC and CAEC. We first examined the expression of endothelial nitric oxide synthase (eNOS), and our immunoblot analysis of protein lysates from P1 to P12 revealed a significant reduction in eNOS levels, which serves as a clear indicator of endothelial dysfunction (Figure 3A).

To investigate whether ES might also modulate the enzymatic activity of eNOS, we continued with our analysis by examining the levels of tetrahydrobiopterin (BH4), a critical cofactor for eNOS-induced NO production. BH4 availability plays a pivotal role in facilitating NO production, while its oxidation to dihydrobiopterin (BH2) primarily contributes to eNOS-mediated induction of superoxide production, the main reactive oxygen species related to the endothelial inflammatory response. Consequently, the BH2/BH4 balance serves as a valuable indicator of NO vs. superoxide generation by eNOS. Our findings align with the observed changes in eNOS expression, as shown by a significant and progressive increase in the BH2/BH4 ratio over time in both endothelial cell types, suggesting that besides eNOS inhibition, replicative ES-induced eNOS enzymatic inhibition may contribute to ES-induced oxidative stress (Figure 3B).

GTP cyclohydrolase (GTPCH), the rate-limiting enzyme in BH4 synthesis, was next assessed in response to ES, unveiling a significant reduction in BH4 over time in both HAEC and CAEC (Figure 3C), while, additionally, BH2 can replenish BH4 through a process known as the salvage pathway in which the dihydrofolate reductase (DHFR) enzyme is implicated. However, in response to ES, DHFR levels were also reduced (Figure 3D). When considered collectively, our data strongly support that ES in HAEC and CAEC leads to significant endothelial dysfunction (ED) through a notable reduction in NO production.

### 2.3. ES Induced Uncoupling of eNOS in HAEC and CAEC

**Figure 4:**
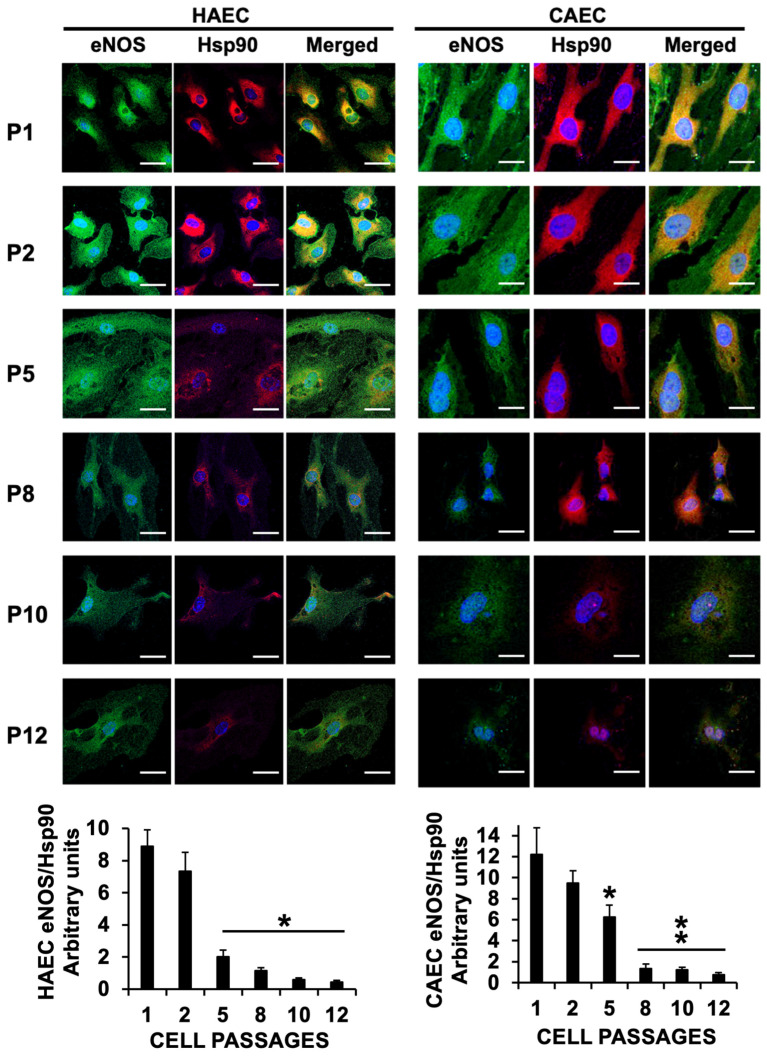
Cell passaging induces eNOS uncoupling from Hsp90 in senescent cells. Representative confocal microscopy of eNOS (FITC, green) and Hsp90 (Alexa 647, red) in HAEC and CAEC from P1 to P12. Merged panels show co-localization of both signals (yellow). N = 3 by triplicate. Mean ± SD. * *p* < 0.01 vs. selected passages (HAEC). * *p* < 0.05, ** *p* < 0.01 vs. selected passages (CAEC). Scale bars, 25 µm.

**Figure 5:**
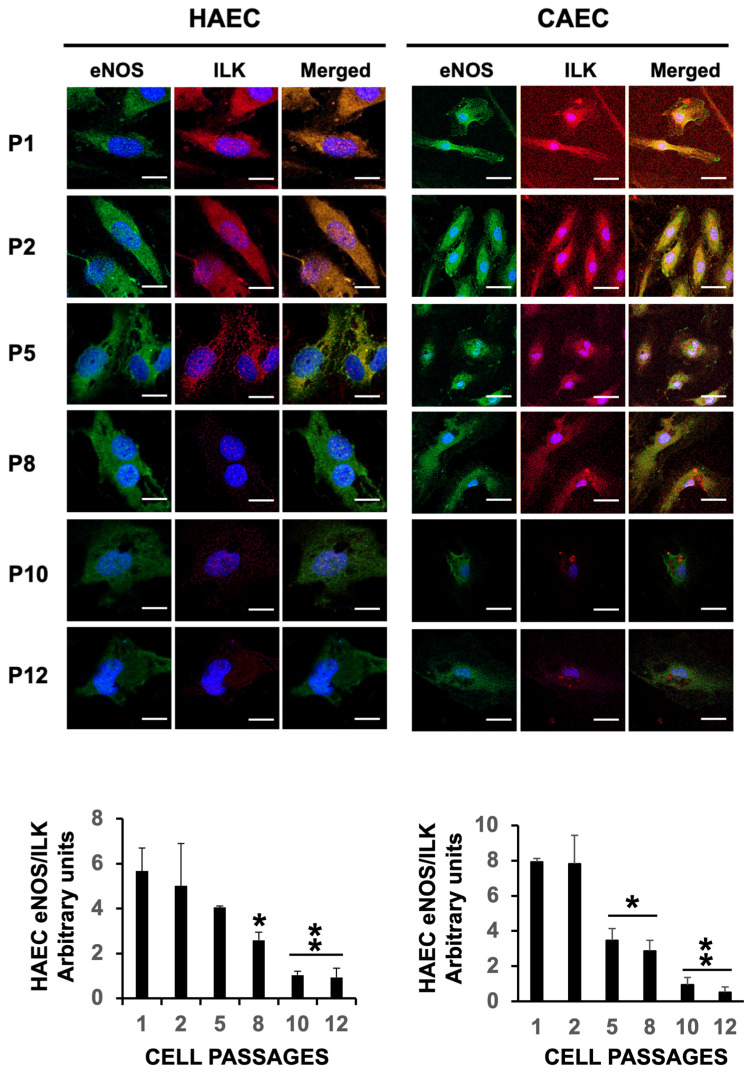
Cell passaging induces eNOS uncoupling from ILK in senescent cells. Representative confocal microscopy of eNOS (FITC, green) and ILK (Alexa 647, red) in HAEC and CAEC from P1 to P12. Merged panels show co-localization of both signals (yellow). N = 3 by triplicate. Mean ± SD. * *p* < 0.05 P1 vs. P8. ** *p* < 0.01 P1 vs. P10, P12 (HAEC). * *p* < 0.01 P1 vs. P5, P8. ** *p* < 0.001 P1 vs. P10, P12 (CAEC). Scale bars, 25 µm.

The data presented above strongly suggest that ES prevents eNOS from enzymatically producing NO. We and others reported that eNOS uncoupling, rather than promoting NO production, leads to the synthesis of eNOS-induced superoxide production in endothelial cells [16]. Besides BH4, eNOS relies on its interaction with the Hsp90 heat shock protein and Interleukin-linked kinase (ILK), which actively participate in the endothelium-dependent vasomotor response, as we have previously described [3]. Confocal microscopy shows a progressive reduction in the expression of eNOS after cell passage 5, as previously shown by immunoblot (Figure 3A), along with a reduction in the levels of ILK and Hsp90. Notably, eNOS co-localization with Hsp90 and ILK markedly decreased after passage 2 (Figure 4 and Figure 5, respectively), providing evidence of eNOS downregulation and eNOS uncoupling during ES.

### 2.4. eNOS Uncoupling Led to Oxidative Stress in Senescent Endothelial Cells

**Figure 6:**
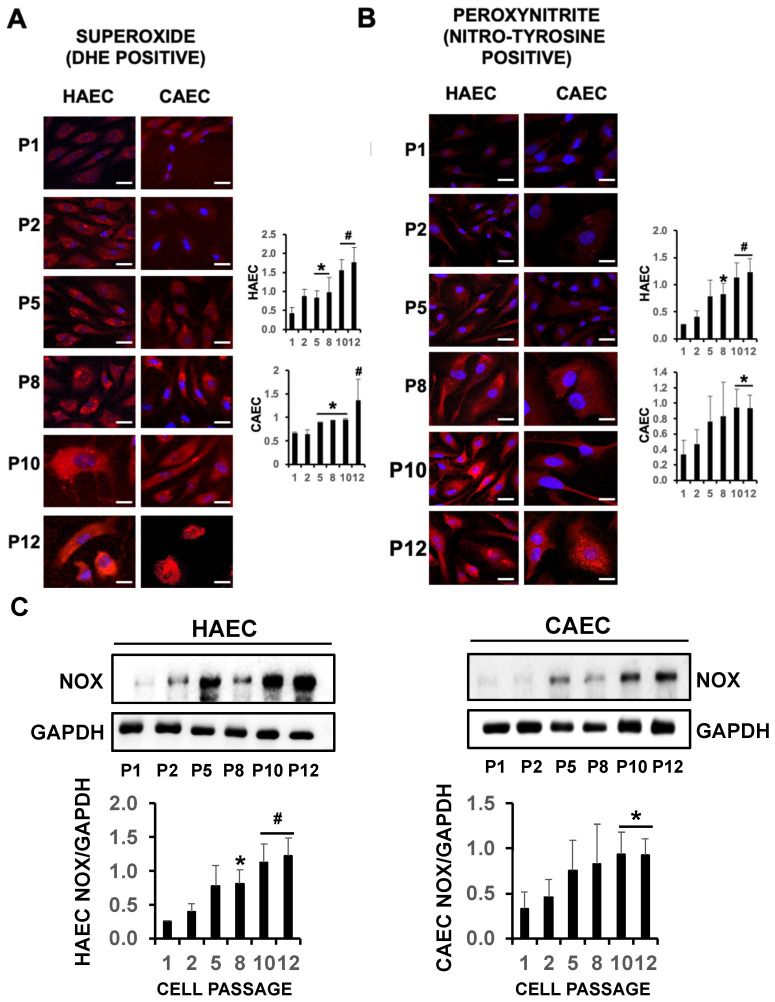
ES induces oxidative and nitrosative stress. Representative confocal microscopy detection of superoxide (**A**) and peroxynitrite (**B**) radicals in HAEC and CAEC from P1 to P12. (**C**) Representative immunoblot detection NOX in HAEC and CAEC from P1 to P12 (N = 3 mean by triplicate ± SD. * *p* < 0.05 P1 vs. indicated passages. ^#^ *p* < 0.01 P1 vs. indicated passages). Scale bars, 25 µm.

Uncoupling of eNOS induces oxidative stress in senescent endothelial cells, as demonstrated by a significant increase in DHE-positive staining from passages 5 to 12 compared to passage 1, indicative of superoxide production in HAEC and CAEC subjected to ES (Figure 6A). Additionally, strong cytotoxic peroxynitrite formation (ONOO^−^) was also evidenced by increased nitrotyrosine positive staining from cell passaging 8 to 12, with respect to cell passage 1, indicative of cytotoxicity and cell death (Figure 6B). Furthermore, eNOS, when uncoupled from ILK and Hsp90, shifts its enzymatic activity from nitric oxide (NO) to superoxide anion. This may explain the observed increase in superoxide radicals in response to ES. Additionally, and in contrast to eNOS, NADPH oxidase levels were also elevated (Figure 6C), supporting the notion that oxidative stress induced by ES arises from a combination of factors, including inhibition of eNOS expression as well as the heightened superoxide production by uncoupled eNOS, and NADPH oxidase in endothelial cells, which may help to explain the negative effect on BH4 availability.

Collectively, our data suggest that endothelial senescence (ES) serves as a catalyst for endothelial dysfunction (ED) and the initiation of inflammatory events, which may include cardiovascular disease.

## 3. Discussion

In this manuscript, we present data that strongly suggest endothelial senescence as a significant cause of endothelial dysfunction, a critical step in developing cardiovascular diseases. In a replicative senescence assay, we observed a positive correlation between oxidative stress and eNOS uncoupling, along with increased oxidation of tetrahydrobiopterin BH4 to BH2. The decreased ILK expression led to superoxide production by eNOS and protein tyrosine nitration. These key events trigger an endothelial inflammatory response, as evidenced by the expression of CyPA together with its receptor EMMPRIN.

Aging is a major factor that prevents nitric oxide bioavailability, a leading cause of endothelial dysfunction (ED), and a key hallmark for the onset and progression of cardiovascular complications including atherosclerosis [8,17]. Endothelial senescence (ES) is an age-related condition associated to ED, a key factor in the early stages of atherosclerosis [18]. However, the factors that trigger endothelial senescence (ES) are still poorly understood. It has been suggested that alterations in the endothelial redox balance are critical in the transition between health and disease. In fact, others have found that the GSSG/GSH ratio may regulate biological aging [19], and the release of oxidized extracellular vesicles induced the binding of human ECs to monocytes [20]. Here, we found that uncoupling of eNOS from the complex with ILK and Hsp90 was associated with increased oxidative and nitrosative stress. This involves changes in the redox state of biopterins shifting from BH4 to oxidized BH2, two metabolites that can be easily measured in blood tests.

On its dimeric form, eNOS-induced NO production fine tunes vascular tone [21,22], in a way to prevent endothelial dysfunction, while actions ending on eNOS configuration to its monomeric state, including the uncoupling from Hsp90 [23] and/or ILK [3,8], prevent eNOS-mediated production of NO, and contribute to the synthesis of superoxide and peroxynitrite radicals [24]. Here, we found that replicative ES correlates not only with a significant reduction in eNOS levels but also with a progressive eNOS uncoupling from Hsp90 and ILK.

We previously demonstrated that eNOS forms a complex with Hsp90 and ILK, which regulates endothelial production of NO and, thus, vascular tone [8]. Therefore, any stimuli that reduce or inhibit ILK could potentially increase oxidative stress. Under these conditions, eNOS will contribute to oxidative stress by producing significant amounts of superoxide anion [3]. Here, we have found a correlation between ES and a decreased expression of eNOS, as well as the uncoupling of eNOS, suggesting that ES may promote the development of ED. Therefore, aging-related ES may create an environment that promotes ED and the subsequent inflammatory response in the onset of CVD [25].

The data presented here may highlight the importance of differentiating between vascular age and chronological age to explain age-related differences in the occurrence of cardiovascular complications [26,27] in which ED may play a significant role. We found that the redox state of eNOS cofactor biopterin was changed from BH4 to oxidized BH2 during ES. A simple blood test measuring circulating BH2 levels could serve as a predictive biomarker for distinguish between chronological and vascular aging. This test might help provide a precise monitoring of selected high-risk patients for future cardiovascular complications, including atherosclerosis, as experimental evidence has already linked increased BH2 levels to the occurrence of peripheral vascular disease [10].

BH4 deficiency affects eNOS uncoupling in various ways. As we mentioned above, eNOS functions as a dimer, and the presence of BH4 is crucial for maintaining this dimeric structure. When BH4 levels are sufficient, they help stabilize the dimer, allowing eNOS to produce NO efficiently. An inadequate BH4 supply causes a shift towards eNOS to a monomeric stage leading to eNOS uncoupling and the production of superoxide instead of NO, which indeed contributes to an additional increase in the oxidative disruption of the dimeric eNOS complex [28].

BH4 depletion also increases eNOS uncoupling by promoting oxidative stress-dependent protein kinase activity. This includes oxidative stress-induced Protein Kinase C phosphorylation at Thr495, which may contribute to uncoupling and superoxide production by eNOS [29].

BH4 is critical in managing the flow of electrons within the eNOS enzyme. It facilitates the transfer of electrons from the NADPH (a reducing agent) to L-arginine, leading to the production of NO. In the absence of BH4, this electron transfer becomes inefficient, resulting in the reduction of molecular oxygen directly to superoxide rather than being used to produce NO, and the oxidative stress promotes the uncoupling of eNOS. In addition, BH4 itself has antioxidant properties and can scavenge reactive oxygen species, including superoxide. This scavenging ability helps protect the eNOS enzyme from oxidative damage, which might otherwise contribute to uncoupling.

The relevance of BH4 deficiency/eNOS uncoupling in the onset and progression of cardiovascular complications, including atherosclerosis, hypertension hypercholesterolemia, or heart failure, has been extensively investigated [30,31,32]. In addition, super oxygenation after percutaneous coronary intervention often leads to vessel wall dysfunction, mostly mediated by the inability of eNOS-induced NO production, in which excessive oxidative conditions may prevent BH4 bioavailability [33,34,35,36,37]. As shown here, replicative senescence also contributes to the depletion of BH4 on its oxidated form BH2. Taken together, we propose to inhibit BH4 oxidation/degradation as a key strategy to prevent ES and subsequent ED.

GTP cyclohydrolase I (GTPCH) is a rate-limiting enzyme during de novo synthesis of BH4, and therefore, GTPCH may play a role in preventing ES by preserving the production of endothelial NO synthesized by dimeric eNOS. GTPCH catalyzes the conversion of GTP to 7,8-dihydroneopterin triphosphate, the first and committed step in the BH4 biosynthetic pathway [38]. Indeed, others found that GTPCH reduction contributes to aging-associated endothelial dysfunction, while overexpression restores age-associated endothelial dysfunction in vivo [12] and prevents age-related microvascular dysfunction [11]. Here, we also found that replicative senescence progressively imbalances the ratio NO/(O_2_^−^ and ONOO^−^), preventing the “de novo” synthetic GTPCH-induced BH4 pathway by reducing the levels of GTPCH.

The molecular structure of human GTPCH1 contains zinc ions to preserve a ring complex between Cys-110, Cys-181, and His-133 in each active site of the enzyme. Therefore, anything that may release zinc from the active site has the potential to inhibit GTPCH enzymatic activity, and, subsequently, BH4 production. Potential oxidants may include Hypochlorous oxide (HOCl) and peroxynitrite (ONOO^−^) [39]. Given the observed increase in peroxynitrite-induced protein tyrosine nitration in HAEC and CAEC, it is possible to speculate that peroxynitrite might also reduce BH4 levels. This aligns with other reports indicating that incubation of endothelial cells with peroxynitrite induces BH4 deficiency, at least in part by increasing the proteasome-dependent degradation of GTPCH [40].

The levels of BH4 in endothelial cells can also be preserved through the “salvage” pathway, where the enzyme dihydrofolate reductase (DHFR) reduces BH2 to help restore BH4 levels. This pathway has been linked to the beneficial effects of PPAR on endothelial cells, as PPAR prevents endothelial dysfunction by increasing DHFR activity [41]. Similar to GTPCH, our data showed that DHFR expression is reduced during replicative senescence, which further contributes to endothelial dysfunction by limiting BH4 availability.

Vascular tone is regulated by endothelial cells through eNOS-mediated production of low levels of NO. However, inflammation induces the expression of another NOS isoform, the inducible nitric oxide synthase (iNOS), in inflammatory cells and smooth muscle cells, leading to the production of high levels of NO. Extensive iNOS-mediated NO formation has been linked to the generation of harmful oxidative products and the progression of atherosclerosis [42]. We demonstrated that iNOS-induced NO production (not from eNOS) promotes eNOS uncoupling that leads to lysosomal-mediated ILK degradation [8], providing for the first-time molecular evidence about the negative effect of excessive NO/iNOS on ILK stability, by inducing the internalization and molecular degradation of ILK in lysosomes. Therefore, we propose that preventing ES may serve as an initial barrier to inhibit the inflammatory response that induces high levels of NO through the expression of iNOS, thereby contributing to the uncoupling of eNOS.

The use of in vitro cell culture models has significantly advanced our understanding of the molecular pathways governing vascular changes in health and disease. For instance, human and bovine aortic endothelial cell (HAEC and BAEC) cultures have been instrumental in revealing, besides a constitutive gene expression, how molecules like TGF-beta and IL-6 regulate eNOS under certain pathophysiological conditions [43]. Similarly, murine aortic endothelial cells (MAECs) have demonstrated the importance of ILK in forming a complex with eNOS to prevent eNOS uncoupling-induced oxidative stress [3] and elucidated the mechanism by which inflammatory responses increase plaque burden by promoting eNOS uncoupling through lysosomal-induced ILK degradation [8].

We propose that oxidative stress-induced eNOS uncoupling is responsible for ES in the vascular endothelium, making strategies aimed at preventing this effect potential pharmacological targets for treating cardiovascular complications. Pharmacological agents that have been explored for preventing eNOS uncoupling include:

- **Antioxidants** like vitamin C, vitamin E, N-acetylcysteine (NAC), or the polyphenol resveratrol can help reduce oxidative stress, improve eNOS function, and increase NO bioavailability [44]. Moreover, ascorbic acid prevents eNOS uncoupling by promoting Ser1177 phosphorylation while preventing Thr455 phosphorylation, which induces the uncoupling of eNOS [45].
- **L-arginine Supplementation.** Cardiovascular risk factors are often increased with L-Arg deficiency [46]. However, controversial results and even detrimental effects of L-arginine supplementation were also reported [47].
- **Renin–angiotensin–aldosterone inhibitors.** Angiotensin II activates NADPH oxidases through AT1 receptor stimulation. Therefore, drugs interfering with the renin–angiotensin–aldosterone system reduce vascular oxidative stress and improve NO bioactivity. Renin inhibitors like Aliskiren prevent NADPH oxidase and increase the levels of BH4, eNOS, and eNOS activity by the induction of eNOS Ser1177 phosphorylation [48,49]. In this regard, ACE inhibitors also prevent eNOS uncoupling by increasing BH4 levels and decreasing nitrotyrosine. Similarly, aldosterone inhibitors like Eplenorone also reduce NADPH oxidase-mediated BH4 oxidation, thus preventing the uncoupling of eNOS [50].
- **Statins** can improve eNOS function and reduce oxidative stress, potentially by eNOS uncoupling in cardiovascular disease by elevating vascular BH4 levels through an increase in the expression of GTPCH1, and inhibit peroxynitrite formation by phorbol ester-stimulated superoxide formation [51,52].
- **Beta blockers.** Nebivolol prevents eNOS uncoupling by reducing oxidative stress through NAPH oxidase inhibition [53]. Interestingly, Nebivolol, but no other betablockers, exerts this effect, and hence, further research is required to fully elucidate the precise mechanism of action.
- **Beta3-adrenergic agonists** like CL316243 is shown to increase eNOS recoupling by reducing oxidative stress and eNOS glutathionylation, a main source of eNOS uncoupling, in pulmonary arterial hypertension [54].
- **PDE5 inhibitors** including Sildenafil, may prevent eNOS uncoupling by preventing the breakdown of cyclic GMP, which might help in maintaining eNOS function [55].
- **Metformin and GLP1 receptor agonists.** Pharmacological treatment of diabetes mellitus includes the administration of Metformin and, more recently, GLP1 analogs with significant results. Metformin recoupled eNOS by upregulating GTPCH1 and BH4 levels and attenuated the upregulation of p47-phox [56], while the GLP1 analog liraglutide has shown a strong capacity of eNOS uncoupling prevention by reducing oxidative stress and S-glutathionylation and increasing NO bioavailability [57].
- **BH4 and Sepiapterin** are effective treatments for cardiovascular conditions, including hypertension, atherosclerosis [58], coronary artery disease [59], diabetes [60], and aging. However, in some cases, the beneficial effect goes beyond NO synthesis [61]. A key limitation of BH4 treatment is the rapid oxidation in the bloodstream, which significantly reduces its effectiveness as a therapeutic tool. To overcome this limitation, others have recently encapsulated BH4 into nanoparticles as a promising tool, showing the efficacy after delivery in NO bioavailability and restoring endothelial function in diabetic rats [62].
- **Folic acid** has been shown to improve cardiovascular health, at least by inducing BH4-dependent eNOS activation. Therefore, folic acid supplements may be effective in preventing the uncoupling of eNOS in cardiovascular complications [63].

The complex interactions between vascular endothelial cells and other cell types in vivo necessitate further investigation to validate these findings. For example, we have reported the in vivo role of ILK in preventing eNOS uncoupling and its effect on atherosclerosis [3], as well as the cardioprotective effects of the eNOS/ILK complex in preventing coronary microvascular dysfunction [7]. Additionally, the role of ILK in human valve endothelial cells in preventing calcific aortic valve disease has been highlighted [19]. These findings underscore the need for in vivo studies to fully understand the intricate dynamics of vascular endothelial cells in their natural tissue environment.

In this study, we used an in vitro replicative senescence model, where cell senescence is induced by telomere attrition following repeated cell divisions. This and other in vitro models offer the advantage of a controlled and reproducible environment to investigate the cellular and molecular changes associated with senescence. However, in vitro studies have significant limitations, such as the reliance on immortalized cell cultures, which may not accurately represent the senescence processes of primary cells, and the absence of in vivo data to support conclusions. To address these concerns, we employed primary aortic and coronary endothelial cells in our replicative senescence model. Nonetheless, in vitro models do not capture the complexity of tissue interactions in the senescence process, the long-term effects associated with aging, or the impact of epigenetics on endothelial aging, regardless of chronological age [64].

In summary, vascular endothelial cell senescence is a major event that may lead to aging-related cardiovascular complications. In this context, assessing blood-circulating BH2 levels may offer a convenient and cost-effective procedure to distinguish between chronological and vascular aging, thereby enabling a more effective selection of patients, even at a subclinical stage.

## 4. Materials and Methods

### 4.1. Reagents and Equipment

The NOX2 (ab129068), DHFR (ab124814), CypA (ab41684), EMMPRIN (ab64616), MMP9 (ab38898), MMP13 (ab39012), GAPDH (ab22555), β-Tubulin (ab6046), and Senescence Detection Kit (ab65351) reagents were from Abcam (Cambridge, UK). Anti-Nitrotyrosine (#06-284) was from Merck Milipore (Burlington, MA, USA). The eNOS (SC-654) antibody was from Santa Cruz Biotechnology (Santa Cruz, CA, USA). The Hsp90 (MA1-10892), Dihydroethidium (Hydroethidine) (D11347), and GTP-Ciclohidrolase (PA5-50508) antibodies were from Thermo Fisher Scientific (Waltham, MA, USA). The ILK (#3862) antibody was from Cell Signaling (Danvers, MA, USA). The ELISA BH4 Kit (MBS733839) was from MyBiosource (San Diego, CA, USA). The ELISA BH2 Kit (abx354212) was from Abbexa (Sugar Land, TX, USA). The Primary Human Aortic Endothelial Cells (HAECs) were from Science Cell (#6100, Carlsbad, CA, USA). The Primary Human Coronary Artery Endothelial Cells (CAECs) (H6093) were from Cell Biologics (#H6093, Cell Biologics, Chicago, IL, USA). ATCC and the distributors have guaranteed that both the HAEC and CAEC primary cells are negative for HIV-1, HBV, HCV, mycoplasma, bacteria, yeast, and fungi.

### 4.2. HAEC and CAEC Cell Culture

The HAEC and CAEC primary cells were cultured in Endothelial Cell Medium (#1001) supplemented with 10% fetal bovine serum (FBS), 5 mg/mL penicillin/streptomycin, and Endothelial Cell Growth Supplement (ECGS) from Science Cell (Carlsbad, CA, USA). The cells were incubated at 37 °C in a humidified atmosphere of 5% CO_2_ and 95% oxygen

### 4.3. Replicative Senescence Assay: SA-β-Gal Staining

Aging-related senescence is associated with several morphological, genetic, epigenetic, and biochemical changes. Notably, telomeres shorten with age in living organisms, and this effect is also observed during in vitro cell culture replication, leading to genetic instability and a phenomenon known as replicative senescence.

Replicative senescence was assessed by detecting SA-β-galactosidase activity. While many mammalian cells express lysosomal β-galactosidase at pH 4, senescent cells allow enzymatic detection at pH 6, making this a useful method for identifying aged cells. Endothelial senescence was evaluated using a Senescence Detection Kit (Abcam) following the manufacturer’s instructions. In brief, cells were fixed with the manufacturer’s Fixative Solution for 10 min, washed with PBS buffer, and incubated with the manufacturer’s staining solution mix for 1 h. Cells were washed with PBS, and images were captured at 40× magnification with a Leica DMi1 bright field microscope. Photomicrograph sections were analyzed for Blue-positive cell quantification with ImageJ, image analysis software, version 1.54 (National Institutes of Health, Bethesda, MA, USA).

### 4.4. Confocal Microscopy

Detection of eNOS (FITC, green) and Hsp90/ILK (Alexa 647, red) was visualized by confocal microscopy in HAECs and CAECs grown on cover slides. Cells were fixed with cold methanol and incubated with 3% BSA in PBS for 1 h. Covers were incubated with primary antibodies (diluted 1:500 in PBS) overnight at 4 °C, washed with PBS, and incubated with the corresponding secondary conjugated antibodies for 1 h at room temperature. The fluorescence-conjugated secondary antibodies, swine anti-goat-FITC (excitation wavelength, 488 nm; emission wavelength, 515 nm), and donkey anti-mouse Alexa 647 (excitation wavelength, 650 nm; emission wavelength, 665 nm) were used. Cell nuclei were detected with Hoechst (excitation wavelength, 405 nm; emission wavelength, 424 nm). Images were captured at 60× magnification using a Leica TCS SP5 confocal microscope (Wetzlar, Germany), and analyzed with ImageJ, image analysis software (National Institutes of Health, Bethesda, MA, USA).

### 4.5. Immunoblotting

Protein lysates were extracted from HAECs and CAECs with RIPA buffer to measure the levels of eNOS, GTP cyclohydrolase, DHFR, NADP Oxidase 2 (NOX2), CypA, EMMPRIN, Matrix Metalloproteinase 9 (MMP9), and Matrix Metalloproteinase 13 (MMP13), as previously described [15]. In brief, proteins were separated by SDS-PAGE and transferred to PVDF membranes. The membranes were blocked with 3% BSA and washed in 25 mM Tris, 150 mM NaCl, 0.05% Tween-20, pH 7.4 (T-TBS), incubated with the corresponding primary antibodies for one hour at room temperature. Subsequently, the membranes were washed 3 times with T-TBS and incubated with horseradish peroxidase-conjugated secondary antibodies 1:3000 for one hour for protein detection by chemiluminescence. GAPDH was used as loading control [7].

### 4.6. Quantification of BH4 and BH2 Levels

BH4 and BH2 were determined using a commercially available ELISA, as recommended by the manufacturer.

### 4.7. Statistical Analysis

Statistical analysis was conducted using SPSS 22.0 (SPSS Inc., Chicago, IL, USA). Results are presented as mean ± standard deviation (S.D.). Significance was determined at the 5% level. When comparisons were made relative to a common control, differences in significance were assessed using analysis of variance followed by Dunnett’s modified *t*-test.

## Data Availability

The original contributions presented in the study are included in the article, further inquiries can be directed to the corresponding author.
